# Circulating AQP4-specific auto-antibodies alone can induce neuromyelitis optica spectrum disorder in the rat

**DOI:** 10.1007/s00401-018-1950-8

**Published:** 2018-12-18

**Authors:** Sophie Hillebrand, Kathrin Schanda, Magdalini Nigritinou, Irina Tsymala, Denise Böhm, Patrick Peschl, Yoshiki Takai, Kazuo Fujihara, Ichiro Nakashima, Tatsuro Misu, Markus Reindl, Hans Lassmann, Monika Bradl

**Affiliations:** 1 Department of Neuroimmunology, Center for Brain Research, Medical University Vienna, Spitalgasse 4, 1090 Vienna, Austria; 2 Clinical Department of Neurology, Innsbruck Medical University, Innsbruck, Austria; 3 Department of Neurology, Tohoku University Graduate School of Medicine, Sendai, Japan; 4 Department of Neurology, Tohoku Medical and Pharmaceutical University, Sendai, Japan

**Keywords:** Neuromyelitis optica, Aquaporin-4, Aquaporin-4-specific antibodies, Lesions, T cells, Kidney

## Abstract

It is well established that the binding of pathogenic aquaporin-4 (AQP4)-specific autoantibodies to astrocytes may initiate a cascade of events culminating in the destruction of these cells and in the formation of large tissue-destructive lesions typical for patients with neuromyelitis optica spectrum disorders (NMOSD). To date, not a single experimental study has shown that the systemic presence of the antibody alone can induce any damage to the central nervous system (CNS), while pathological studies on brains of NMOSD patients suggested that there might be ways for antibody entry and subsequent tissue damage. Here, we systemically applied a highly pathogenic, monoclonal antibody with high affinity to AQP4 over prolonged period of time to rats, and show that AQP4-abs can enter the CNS on their own, via circumventricular organs and meningeal or parenchymal blood vessels, that these antibodies initiate the formation of radically different lesions with AQP4 loss, depending on their mode and site of entry, and that lesion formation is much more efficient in the presence of encephalitogenic T-cell responses. We further demonstrate that the established tissue-destructive lesions trigger the formation of additional lesions by short and far reaching effects on blood vessels and their branches, and that AQP4-abs have profound effects on the AQP4 expression in peripheral tissues which counter-act possible titer loss by antibody absorption outside the CNS. Cumulatively, these data indicate that directly induced pathological changes caused by AQP4-abs inside and outside the CNS are efficient drivers of disease evolution in seropositive organisms.

**Electronic supplementary material:**

The online version of this article (10.1007/s00401-018-1950-8) contains supplementary material, which is available to authorized users.

## Introduction

Aquaporin 4 (AQP4)-specific antibodies (AQP4-abs) are found in the majority of patients with neuromyelitis optica spectrum disorder (NMOSD), a severe autoimmune disease of the central nervous system (CNS) culminating in antibody-mediated destruction of astrocytes. These antibodies are highly pathogenic when they are applied together with complement to spinal cord slices in vitro [60], when they are injected together with complement into the brains of experimental animals [39, 42], or when they are intraperitoneally injected together with encephalitogenic T cells into rodents [4, 23, 35, 36, 43, 53, 57]. However, 24 h after the systemic application of immunoglobulin preparations from NMOSD patients (the so-called NMO-IgGs) or recombinant human AQP4-abs into mice or rats [4, 21, 40], the antibodies were found on astrocytes in the area postrema [40], but not elsewhere in the CNS [4, 21, 40]. Hence, to date, not a single experimental study has shown that the systemic presence of the antibody alone can induce any damage to the CNS. However, some pathological studies indicated that there may be ways, how antibodies can be directly pathogenic, and that the area postrema [37] or the blood–cerebrospinal fluid (CSF) barrier [9] might be primary entry sites for NMO-IgG into the CNS. This clearly demonstrates that there is a pronounced discrepancy between the observations in animal models and human patients. The reason for this could be that the NMO-IgGs used in the animal models did not only contain AQP4-abs, but additionally other immunoglobulins, that the AQP4-abs found in NMO-IgG preparations were polyclonal, with differences in pathogenicity and affinities [7], that the human AQP4-abs suboptimally interact with rat complement [4], or that the animal studies just simply did not reach the perfect time window for CNS injury [4, 21, 40]. For all these reasons, it was of essential and critical importance to make a proof of principle study with an antibody transfer, which may overcome these potential problems. This was done in the current study, using a highly pathogenic, monoclonal antibody [16, 23, 30] with high affinity to AQP4, systemically applied over prolonged period of time.

## Materials and methods

### Animals

7- to 8-week-old Lewis rats and Rowett Nude (RNU) rats were obtained from Charles River Wiga (Sulzfeld, Germany), and were housed in the Decentral Facilities of the Institute for Biomedical Research (Medical University Vienna) under standardized conditions. All applicable international, national, and/or institutional guidelines for the care and use of animals were followed. All procedures performed in studies involving animals were in accordance with the ethical standards of the institution or practice at which the studies were conducted. The experiments were approved by the Ethic Commission of the Medical University Vienna and performed with the license of the Austrian Ministry for Science and Research (GZ: BMBWF-66.009/0136-WF/V/3b/2016, BMBWF-66.009/0107-V/3b/2018, and BMBWF-66.009/0221-V/3b/2018).

### Antibodies and T cells

We used the monoclonal AQP4-specific antibody E5415A [23], termed “AQP4-abs” throughout the manuscript, and control mouse IgG (Sigma, Vienna, Austria), both in a concentration of 1 mg/ml in phosphate-buffered saline (PBS). The T-cell lines specific for myelin basic protein (MBP) and aquaporin 4 (AQP4_268–285_) were essentially produced as described [57].

### Transfer of antibodies

The animals were intraperitoneally (i.p.) injected with 1 mg AQP4-abs or control mouse IgG under isoflurane anesthesia for 1, 2, and 5 consecutive days, and were scored daily for the presence of clinical symptoms. 24 h after the last injection, the animals were killed with CO_2_ and bled to obtain serum for the determination of antibody titers. Then, the rats were perfused with 4% paraformaldehyde (PFA) in PBS. Spinal cords, brains, eyes, and peripheral organs were dissected, postfixed for 24 h in 4% PFA, and embedded in paraffin for histological analysis.

### Transfer of antibodies to animals harboring activated antigen-specific T cells

For the induction of experimental neuromyelitis optica spectrum disorder (ENMOSD), myelin basic protein (MBP)- or AQP4_268–285_-specific T cells were injected i.p. on day 0, followed by an i.p. injection with 1 ml PBS containing 0.5 or 1 mg E5415A on day 4. These animals were killed on days 5/6 after T-cell transfer with CO_2_, perfused with 4% PFA, and dissected. Their tissues were fixed in 4% PFA for additional 18–24 h and embedded in paraffin for histological analysis.

### Titer determinations

Serum was used to determine the titers of AQP4-abs using cell-based assays essentially as described [4, 26], and to determine the concentrations of mouse IgG using the IgG (Total) Mouse Uncoated ELISA Kit (Invitrogen) according to the instructions of the manufacturer, with a 1:500 dilution of rat serum.

### Immunohistochemistry

2–4 μm-thick sections were cut on a microtome. The sections were dewaxed in xylol for 30 min, transferred to 96% ethanol, and incubated in 0.2% hydrogen peroxide for 30 min to block endogenous peroxidase. Then, the sections were rehydrated through a descending ethanol series (96, 70, and 50%), rinsed in distilled water, and subjected to heat-induced antigen retrieval by heating them for 15 min in 0.03% mM protease-type XXIV (for C9neo antibodies) at 37 °C or for 60 min in 10 mM EDTA pH 8.5 (for all the other antibodies) in a conventional household steamer. Subsequently, the sections were rinsed in 0.1 M PBS or Tris-buffered saline (TBS) for 60 min, and exposed to 10% fetal calf serum (FCS) in 1 × DAKO Wash Buffer in PBS for 20 min at room temperature to reduce non-specific background. Then, immunohistochemical stainings were done essentially as described [4], using the following antibodies: polyclonal rabbit anti-rat AQP4 (1:250, Sigma-Aldrich, Vienna, Austria), donkey anti-rat IgG (1:1500, Jackson ImmunoResearch, West Grove, PA, USA), rabbit anti-rat C9neo (1:2000, [34]), polyclonal rabbit anti-cow glial fibrillary acidic protein (GFAP, cross-reactive with rat; 1:3000; DakoCytomation), monoclonal mouse anti-rat ED1 (1:10,000; Thermo Scientific, Vienna, Austria), and monoclonal rabbit anti-human CD3 (cross-reactive to rat CD3, 1:2000, Thermo Scientific). Immunohistochemistry was completed by incubation with corresponding biotinylated secondary antibodies (donkey anti-rabbit, 1:2000, sheep anti-mouse, 1:500, both antibodies from Jackson ImmunoResearch; donkey anti-sheep/goat, 1:200, Amersham GE Healthcare), followed by exposure to avidin-peroxidase complex (1:100 in DB/FCS; Sigma). Enhancement of the CD3 staining was performed using biotinylated tyramine amplification. In case of C9neo, labeling was visualized with the AEC system, in all the other cases with 3,3′ diaminobenzidine-tetra-hydrochloride (DAB, Sigma) containing 0.01% hydrogen peroxide. All sections were counterstained with Meyer’s hematoxylin, dehydrated and mounted in geltol (sections developed with the AEC system) or Eukitt© (Merck, Darmstadt, Germany) (all the other sections).

For the detection of eosinophils, Giemsa staining was made. Briefly, deparaffinised and rehydrated tissue sections were incubated in Giemsa working solution [15 ml Giemsa’s azure eosin methylene blue solution (Merck), 85 ml distilled H_2_O] for 30 min. The tissue was differentiated in distilled H_2_O enriched with drops of pure acetic acid. After washing steps with distilled H_2_O and 96% ethanol, the slides were transferred into ester and sealed with Eukitt©.

## Results

### AQP4-abs can induce NMOSD lesions without T-cell help

**Fig. 1:**
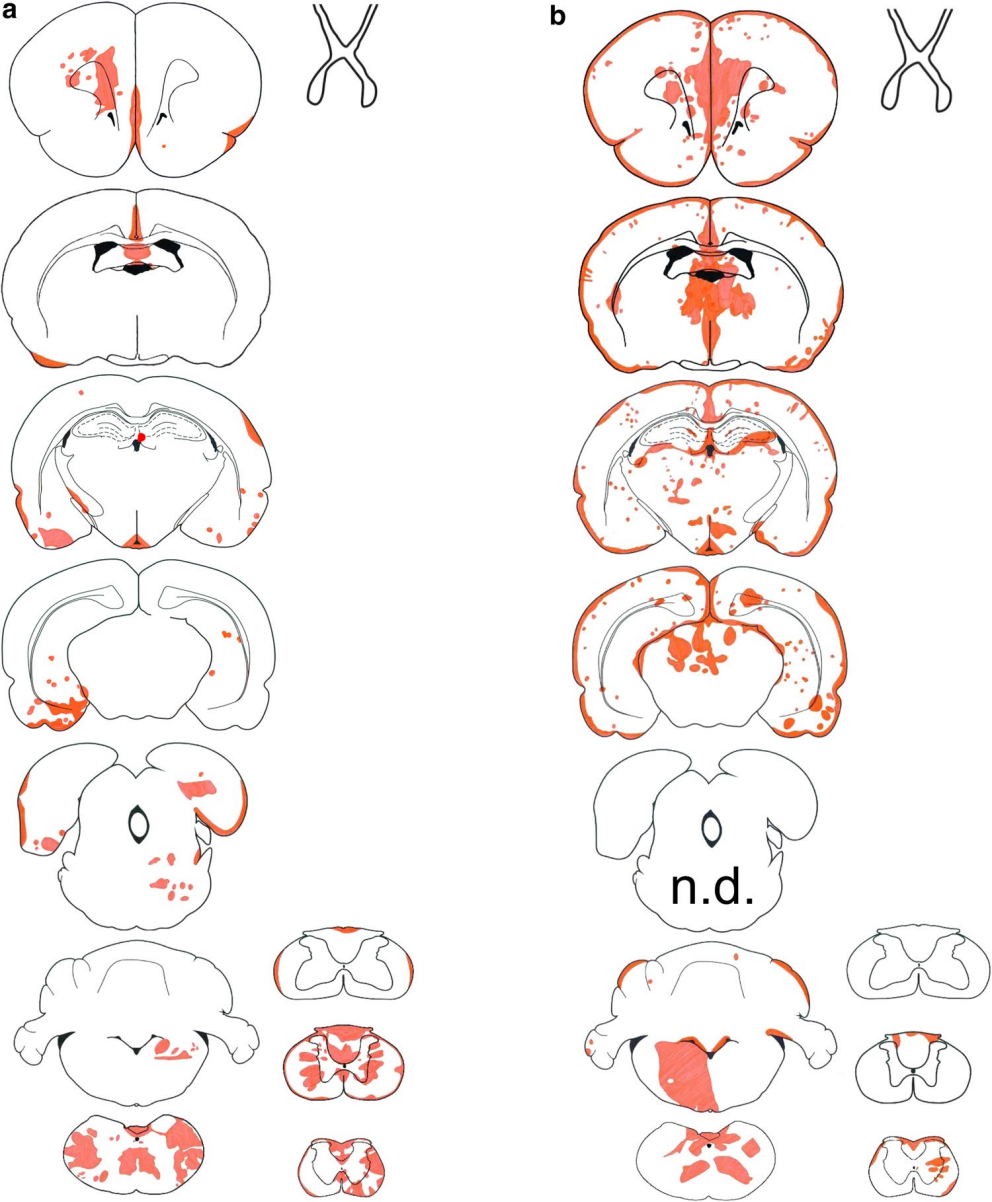
Lesions with aquaporin 4 loss in AQP4-specific antibody-injected rats. Distribution of established lesions with AQP4 loss along the neuraxis, using schemes provided by Paxinos and Watson [33] as guide lines. Shown here are brain and spinal cord [cervical (C1–7), thoracal (T1–10) and lumbar/sacral (L1–S4)] sections, as well as outlines of optic nerve, chiasm, and optic tract of Lewis (**a**, *n* = 5) and RNU rats (**b**, *n* = 5). The animals were analyzed 120 h after daily intraperitoneal injections of AQP4-abs, and the location of each lesion with AQP4 loss was projected in red color into the relevant scheme

In the first 24–48 h after the initiation of AQP4-specific antibody injections into Lewis rats, AQP4 loss was only observed in the area postrema (Table 1). After 120 h, AQP4 loss was noted throughout the brain and spinal cord (Fig. 1), with large inter-individual differences both in location and extent of lesions (suppl. Figure 1), and in clinical symptoms (Table 1). Chiasms and optic nerves did not show lesions with AQP4 loss. All control animals injected with mouse IgG did not show any signs of inflammation or AQP4 loss, and remained healthy (Table 1). Lesions with AQP4 loss (Fig. 1, Table 1) and associated clinical symptoms (Table 1) also developed in athymic RNU rats in which T-cell function is virtually absent [13], and which lack the thymus-dependent T cells implied in the pathogenesis of NMOSD [5, 13].

**Table 1:** Antibody titers/concentrations, neurological symptoms, and location of established lesions with AQP4 loss in Lewis rats (L) and RNU rats (R)

	0 h	24 h αAQP4 ab	48 h αAQP4 ab	120 h αAQP4 ab	24 h mIgG (co)	48 h mIgG (co)	120 h mIgG (co)
Serum total mouse IgG (µg/ml)	0	11.5 (L)	17.2 (L)	45.7 (L)	n.d.	19.0 (L)	51.4 (L)
AQP4-antibody titer (titer, 1:)	n.d.	32,768 (L)	32,768 (L)	851,968 (L) 382,293 (R)	n.d. n.d.	n.d. n.d.	n.d. n.d.
Neurological symptoms	0/5 (L) 0/5 (R)	0/5 (L) 0/5 (R)	0/5 (L) 0/5 (R)	(L): 1/5 excessive salivation (rat correlate for nausea/vomiting behavior [34]); 1/5 problems with balance; 1/5 inability to properly place hind limb while walking; 2/5 clinically normal (R): 2/5 weak; 1/5 seemingly painful sensations in front paws (possible correlate to pain in NMO [3]); 2/5 clinically normal	0/5 (L)	0/5 (L)	0/5 (L)
Lesions (s. cord)	0/4	0/5	0/5	4/5 (L) 3/3 (R)	0/5	0/5	0/5
Lesions (brain)							
Circumventricular organs							
Area postrema	0/4	5/5	5/5	4/4 (L) 5/5 (R)	0/5	0/5	0/5
Eminentia mediana	0/2	0/5	0/4	1/5 (L) 4/5 (R)	0/5	0/3	0/2
Subfornical organ	0/4	0/2	0/0	2/2 (L) 0/0 (R)	0/4	0/4	0/3
Ventricles	0/5	0/5	0/5	3/5 (L) 4/5 (R)	0/5	0/5	0/5
Medulla outside area postrema	0/4	0/5	0/5	2/5 (L) 2/5 (R)	0/5	0/5	0/5
Cerebellum	0/4	0/5	0/5	0/5 (L) 3/5 (R)	0/5	0/5	0/5
Thalamus	0/4	0/5	0/5	0/5 (L) 4/5 (R)	0/5	0/5	0/5
Hypothalamus	0/4	0/5	0/5	0/5 (L) 2/5 (R)	0/5	0/5	0/5
Midbrain/pons	0/4	0/5	0/5	1/5 (L) 1/5 (R)	0/5	0/5	0/5
Temporal lobe	0/4	0/5	0/5	2/5 (L) 3/5 (R)	0/5	0/5	0/5
Striatum	0/4	0/5	0/5	2/5 (L) 4/5 (R)	0/5	0/5	0/5
Lesions (optic system)							
Retina	0/4	0/5	0/5	1/5 (L, uni-lateral) 0/5 (R)	0/5	0/5	0/5
Optic nerves	0/4	0/5	0/5	0/5 (L) 0/5 (R)	0/5	0/5	0/5
Chiasm	0/2	0/3	0/4	0/3 (L) 0/2 (R)	0/2	0/2	0/1
Optic tract	0/4	0/5	0/5	0/5 (L) 0/5 (R)	0/5	0/5	0/5

The CNS was analyzed along the entire neuraxis after daily injections of antibodies against aquaporin 4 (αAQP4) or of murine immunoglobulin (mIgG) used as control (co)

*n.d.* not done

Hence, AQP4-abs can induce NMOSD lesions alone, without the help of T cells.

### AQP4-abs use three different modes of entry into the CNS

#### Antibody entry through circumventricular organs

**Fig. 2:**
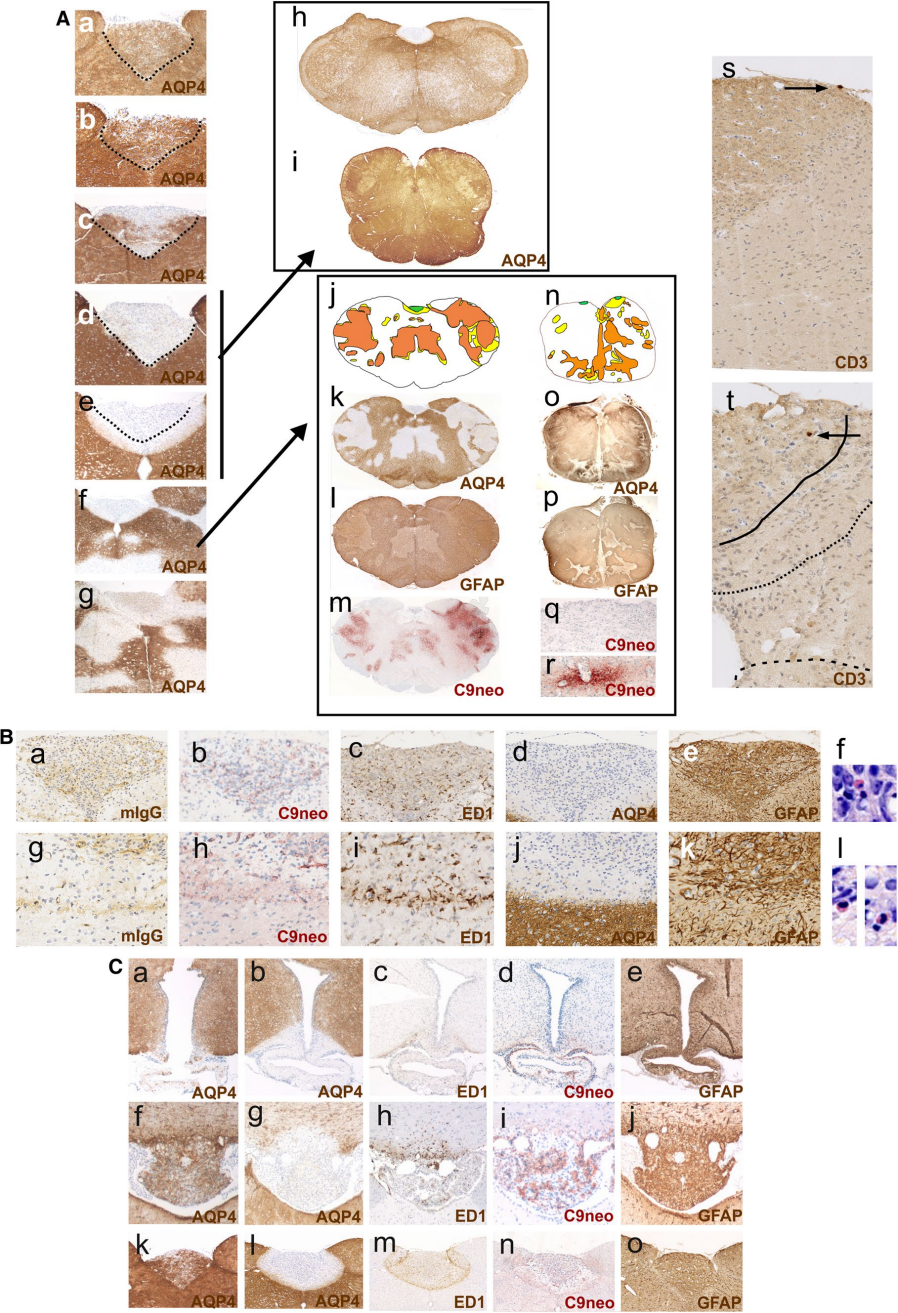
Loss of aquaporin 4-reactivity in circumventricular organs. **A** Time course of AQP4 loss from the area postrema of Lewis rats. AQP4 stainings (brown) of representative area postremae from uninjected animals (**A**a), animals injected daily with mouse control IgG and sacrificed after 120 h (**A**b), and animals injected daily with the monoclonal AQP4-specific murine E5415A IgG and sacrificed after 24 (**A**c), 48 (**A**d), and 120 h (**A**e–**A**g). All sections were counterstained with hematoxylin to reveal nuclei in blue. The dotted black line depicts the outline of the area postremae. **A**h, **A**i In some cases, AQP4 loss at the area postrema may be the only pathological changes observed in the medulla of Lewis rats and human patients. AQP-4 stained cross sections of the medulla of a Lewis rat injected daily with the monoclonal AQP4-specific murine E5415A IgG and sacrificed after 120 h (**A**h) and a patient with NMOSD (**A**i). **A**j–**A**r In other cases, the medulla shows AQP4 loss at the area postrema and additionally also perivascular loss of AQP4 reactivity. Medullary cross sections at the level of the area postrema were made from a Lewis rat injected daily with the monoclonal AQP4-specific murine E5415A antibody and sacrificed after 120 h (**A**j–**A**m), and from an NMOSD patient (**A**n–**A**r). The lesions observed were projected into the corresponding schemes (**A**j, **A**n), with the area postrema shown in green, lesions with loss of AQP4 reactivity in yellow, and areas with loss of GFAP reactivity in orange. The corresponding stainings with α-AQP4 antibodies (brown, loss of AQP reactivity white; **A**k, **A**o), with α-GFAP antibodies (dark brown, loss of reactivity pale brown; **A**l, **A**p), and α-complement C9neo (red; **A**m, **A**q, **A**r) are shown. Counterstaining was done with hematoxylin to reveal nuclei (blue). Note that, in the experimental animal and in the human NMOSD case, the area postrema does not show complement C9neo reactivity (**A**m, **A**q), while perivascular lesions in distance to the area postrema show complement deposition (**A**m, **A**r). T cells are essentially absent from the medullas of experimental Lewis rats injected with the monoclonal AQP4-specific murine E5415A antibody and sacrificed after 48 (**A**s) and 120 h (**A**t). Shown are medullary sections at the level of the area postrema, stained with α-CD3 antibodies (brown). **B** Consecutive histological sections of the medulla of an AQP4-specific antibody-injected Lewis rats, 120 h after the initiation of antibody injection. Shown here are the consequences of AQP4 loss for the area postrema (**B**a–**B**f) and the area subpostrema (**B**g–**B**l). The sections were stained with antibodies specific for murine IgG (**B**a, **B**g), complement C9neo (**B**b, **B**h), ED1 (**B**c, **B**i), AQP4 (**B**d, **B**j), and GFAP (**B**e, **B**k). With exception of complement C9neo, where a positive reaction product is shown in red (**B**b, **B**h), all other antibody reaction products are shown in brown. Counterstaining was done with hematoxylin to reveal nuclei (blue). Eosinophils were visualized by Giemsa staining (**B**f, **B**l; red). **C** Loss of aquaporin 4-reactivity in circumventricular organs and parenchymal lesions with aquaporin 4-loss spreading from these sites. Histological sections of eminentia mediana (**C**a–**C**e), subfornical organ (**C**f–**C**j), and area postrema (**C**k–**C**o) were stained for AQP4 (brown, **C**a, **C**b, **C**f, **C**g, **C**k, **C**l), ED1 (brown, **C**c, **C**h, **C**m), complement C9neo (red, **C**d, **C**i, **C**n), and GFAP (brown, **C**e, **C**j, **C**o). The sections shown derived from control Lewis rats (**C**a, **C**f, **C**k) and from Lewis rats injected daily with the monoclonal AQP4-specific murine E5415A IgG and sacrificed after 120 h. In all sections, counterstaining was done with hematoxylin to reveal nuclei (blue)

The area postrema was the most frequently affected site in all AQP4-specific antibody-injected rats (Figs. 1 and 2, suppl. Figure 1), in line with the presence of fenestrated endothelial cells allowing the passage from antibodies to this circumventricular organ [29]. After 24 h, the loss of AQP4 reactivity in the area postrema was patchy; after 48 h, it was complete (suppl. Figure 2), and after 120 h, the AQP4-abs diffused from the area postrema into the surrounding tissue, causing AQP4 loss beyond the area postrema. The eminentia mediana was less often affected by AQP4 loss (Table 1). However, also at this site, lesions with AQP4 loss and intact GFAP reactivity spread into the surrounding tissue, contained ramified ED1^+^ microglia, and showed a sharp border to areas with intact AQP4 reactivity. Such lesions could also involve the floor of the third ventricle (Fig. 1). We also saw AQP4 loss in the subfornical organs (Fig. 2) and lesions with AQP4 loss adjacent to this site, but did not analyze this further.

#### Antibody entry through meningeal vessels and veins of the Virchow Robin spaces

**Fig. 3:**
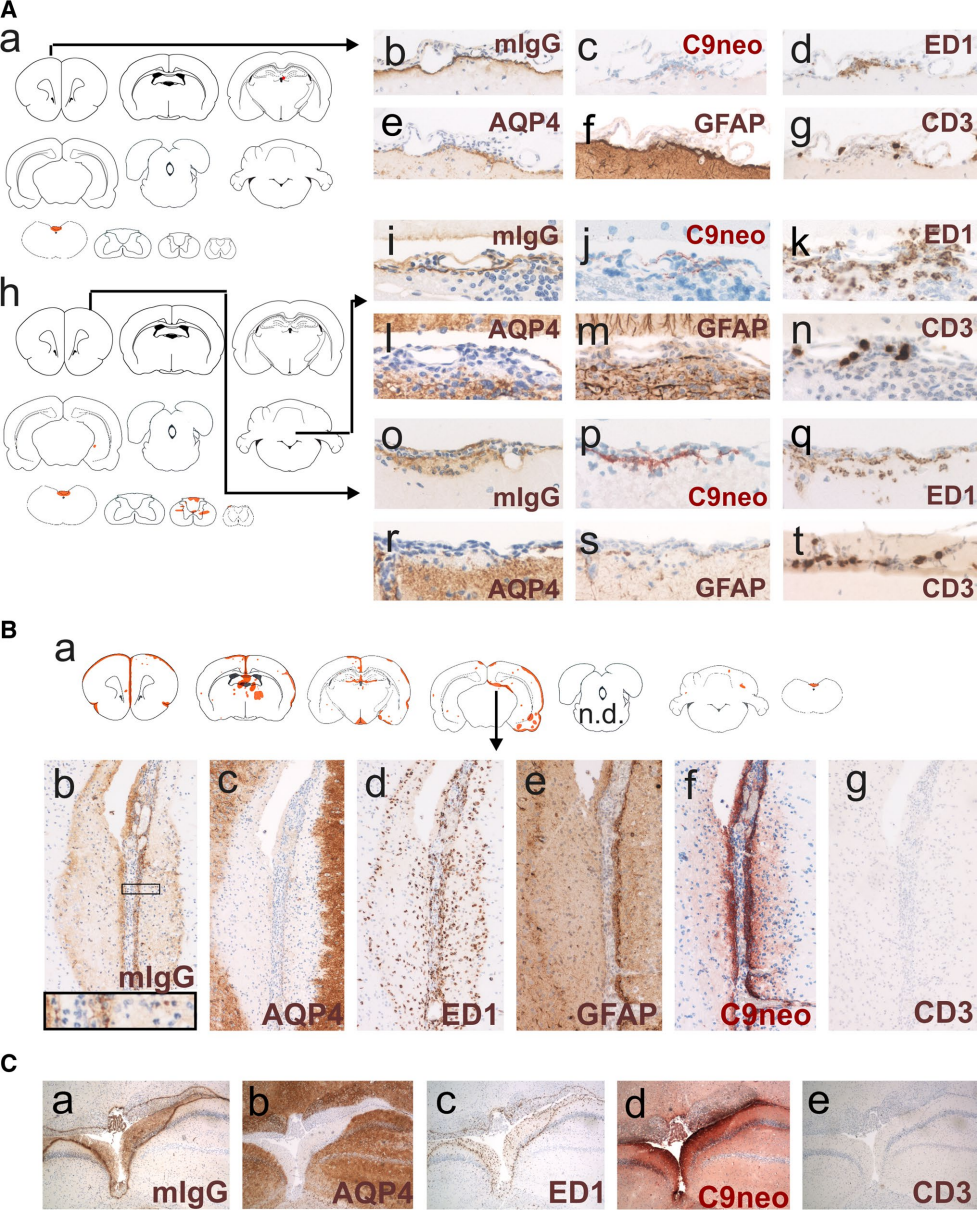
Subpial loss of aquaporin 4-reactivity. **A** Ongoing formation of subpial lesions. Shown first is the distribution of established lesions with AQP4 loss in brain and spinal cord of single Lewis rats (**A**a, **A**h) which had been injected daily with the monoclonal AQP4-specific murine E5415A IgG and sacrificed after 120 h. The location of each established lesion with AQP4 loss was projected in red color into the relevant scheme. The site of the earliest ongoing lesions used for further characterization is indicated by black arrows in CNS. Consecutive sections of these earliest ongoing lesions were then reacted with antibodies against murine IgG (**A**b, **A**i, and **A**o, brown) or against complement C9neo (**A**c, **A**j, **A**p, red), with the ED1 antibody (**A**d, **A**k, **A**q, brown), and antibodies against AQP4 (**A**e, **A**l, **A**r, brown), against GFAP (**A**f, **A**m, **A**s, brown), and against CD3 (**A**g, **A**n, **A**t, brown; please note that, in this picture, parts of the section were folded back). In all sections, counterstaining was done with hematoxylin to reveal nuclei (blue). **B** Characterization of subpial lesion from an RNU rat injected daily with the monoclonal AQP4-specific murine E5415A IgG and sacrificed after 120 h. **B**a Distribution of established lesions with AQP4 loss in this animal is shown along the neuraxis, using schemes provided by Paxinos and Watson [33] as guide lines. Shown here are brain and spinal cord [cervical (C1–7), thoracal (T1–10), and lumbar/sacral (L1–S4)] sections, as well as outlines of optic nerve, chiasm, and optic tract, and the location of each lesion with AQP4 loss was projected in red color into the relevant scheme. The arrow shows the location of the subpial lesion further characterized by stainings with antibodies against murine IgG (**B**b, brown, inlay shows the presence of neutrophils), AQP4 (**B**c, brown), with the ED1 antibody (**B**d, brown), and with antibodies against GFAP (**B**e, brown), complement C9neo (**B**f, red) and CD3 (**B**g, brown; please note the complete absence of T cells). In all sections, counterstaining was done with hematoxylin to reveal nuclei (blue). **C** Characterization of an extensive periventricular lesion formed in an RNU rat which had been injected daily with the monoclonal AQP4-specific murine E5415A IgG and sacrificed after 120 h. Consecutive sections at the level of the third ventricle were reacted with anti-mouse IgG (**C**a, brown), anti-AQP4 (**C**b, brown), ED1 (**C**c, brown), anti-complement C9neo (**C**d, red), and anti-CD3 (**C**e, brown). In all sections, counterstaining was done with hematoxylin to reveal nuclei (blue)

120 h after the initiation of antibody injections, both Lewis and RNU rats developed meningitis, as evidenced by the presence of neutrophils, macrophages, and T cells (in Lewis rats). To learn more about antibody entry at this site, we studied ongoing lesions in the form of subpial microlesions/microinfiltrates in rats which essentially lacked brain lesions apart from area postrema changes. These microlesions/microinfiltrates demonstrated the extravasation of macrophages and T cells from meningeal vessels, with varying levels of antibody diffusion into the adjacent parenchyma, and with complement deposition and ongoing AQP4 loss within the underlying tissue (Fig. 3). From these vessels, antibodies might also access the CSF.

We also observed vessels containing intra- and extraluminal neutrophils entering the CNS parenchyma from the pial surface. Since some of these vessels showed leakage of immunoglobulins into the CNS parenchyma, it is possible that extraluminal neutrophils derive from circulating cells entering the CNS through an open blood–brain barrier (BBB). However, many of the extraluminal neutrophils could also represent meningeal infiltrates, reaching the CSF from meningeal vessels, and the site of lesion formation via CSF and Virchow–Robin spaces.

Cumulatively, these data show antibody entry from the meningeal vasculature into the perivascular parenchyma, and suggest also antibody entry from meningeal vessels into the CSF.

#### Antibody entry through small parenchymal vessels unrelated to the meninges

**Fig. 4:**
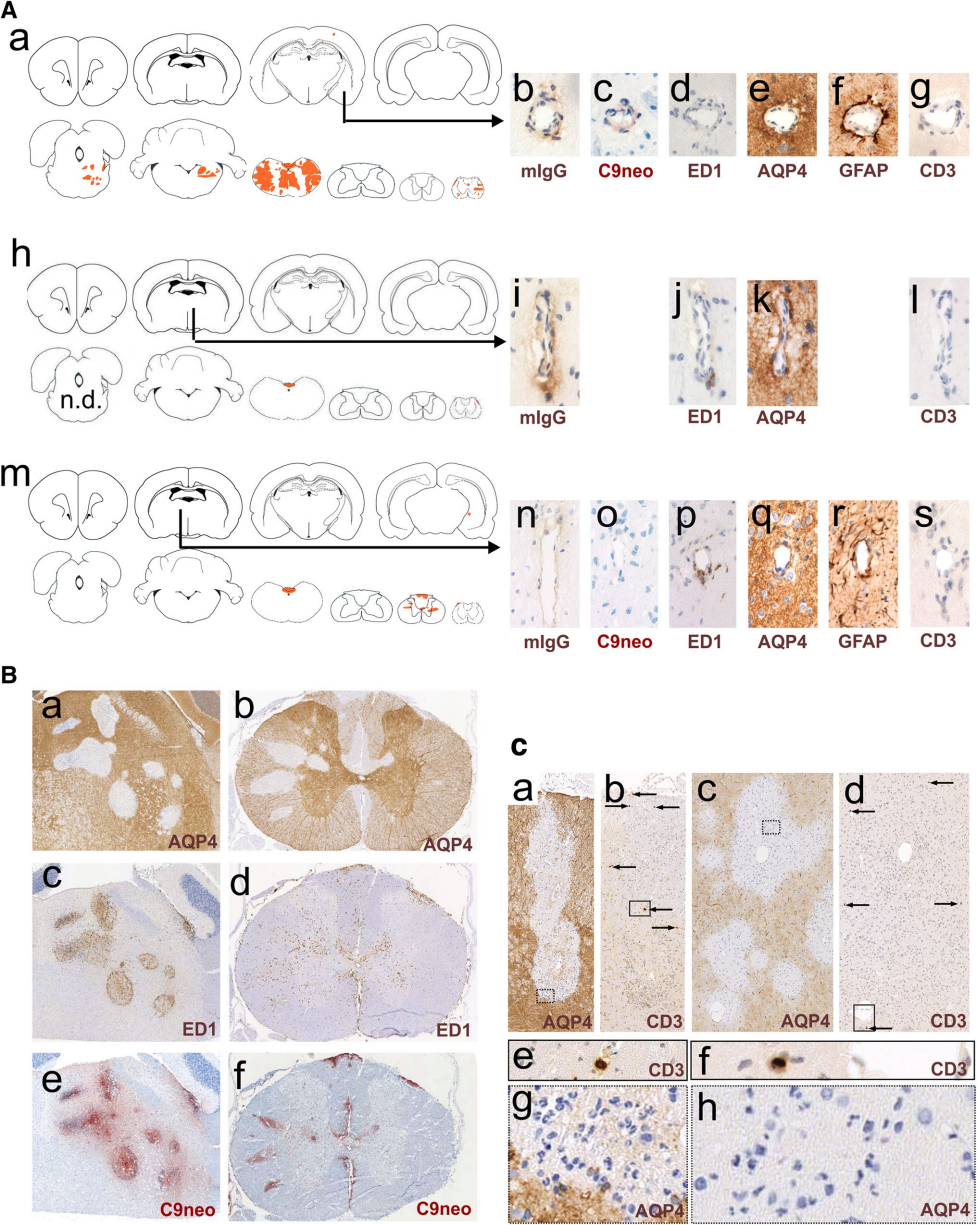
Perivascular loss of aquaporin 4-reactivity. **A** Localization and immunohistochemical characterization of leaky vessels allowing antibody entry into the CNS parenchyma. Shown here is the lesion load of individual animals, projected in red onto brain and spinal cord schemes provided by Paxinos and Watson [33] as guide lines (**A**a, **A**h, **A**m). The ends of the black arrows indicate the exact location of the vessels reacted in consecutive sections with anti-mouse IgG (**A**b, **A**i, **A**n, brown), anti-complement C9neo (**A**c, **A**o, red), ED1 (**A**d, **A**j, **A**p, brown), anti-AQP4 (**A**e, **A**k, **A**q, brown), anti-GFAP (**A**f, **A**r, brown), and anti-CD3 (**A**g, **A**l, as, brown). In all sections, counterstaining was done with hematoxylin to reveal nuclei (blue). **B** Histological characterization of perivascular lesions found in the brain (**B**a, **B**c, **B**e) and spinal cord (**B**b, **B**d, **B**f) of Lewis rats injected daily with the monoclonal AQP4-specific murine E5415A IgG and sacrificed after 120 h. Consecutive sections were stained with anti-AQP4 (**B**a, **B**b; brown), ED1 (**B**c, **B**d; brown), and anti-complement C9neo (**B**e, **B**f; red). Counterstaining was done with hematoxylin to reveal nuclei (blue). **C** Demonstration of neutrophils in and of a near-complete absence of T cells from perivascular lesions. Consecutive lesions derived from spinal cord (**C**a, **C**b, **C**e) and brain (**C**c, **C**d, **C**f) of Lewis rats injected daily with the monoclonal AQP4-specific murine E5415A IgG and sacrificed after 120 h, and were reacted with antibodies against AQP4 (**C**a, **C**c, **C**g, **C**h) and against CD3 (**C**b, **C**d, **C**e, **C**f). The squares with dotted lines in **C**a and **C**c outline areas shown in higher magnification in **C**g and **C**h, respectively, to demonstrate the presence of neutrophils. The arrows in **c**b and **c**d point to CD3-positive T cells, and the squares with straight lines shown in **C**b and **C**d indicate lesion areas shown in higher magnification in **C**e and **C**f, respectively, to show single T cells

**Fig. 5:**
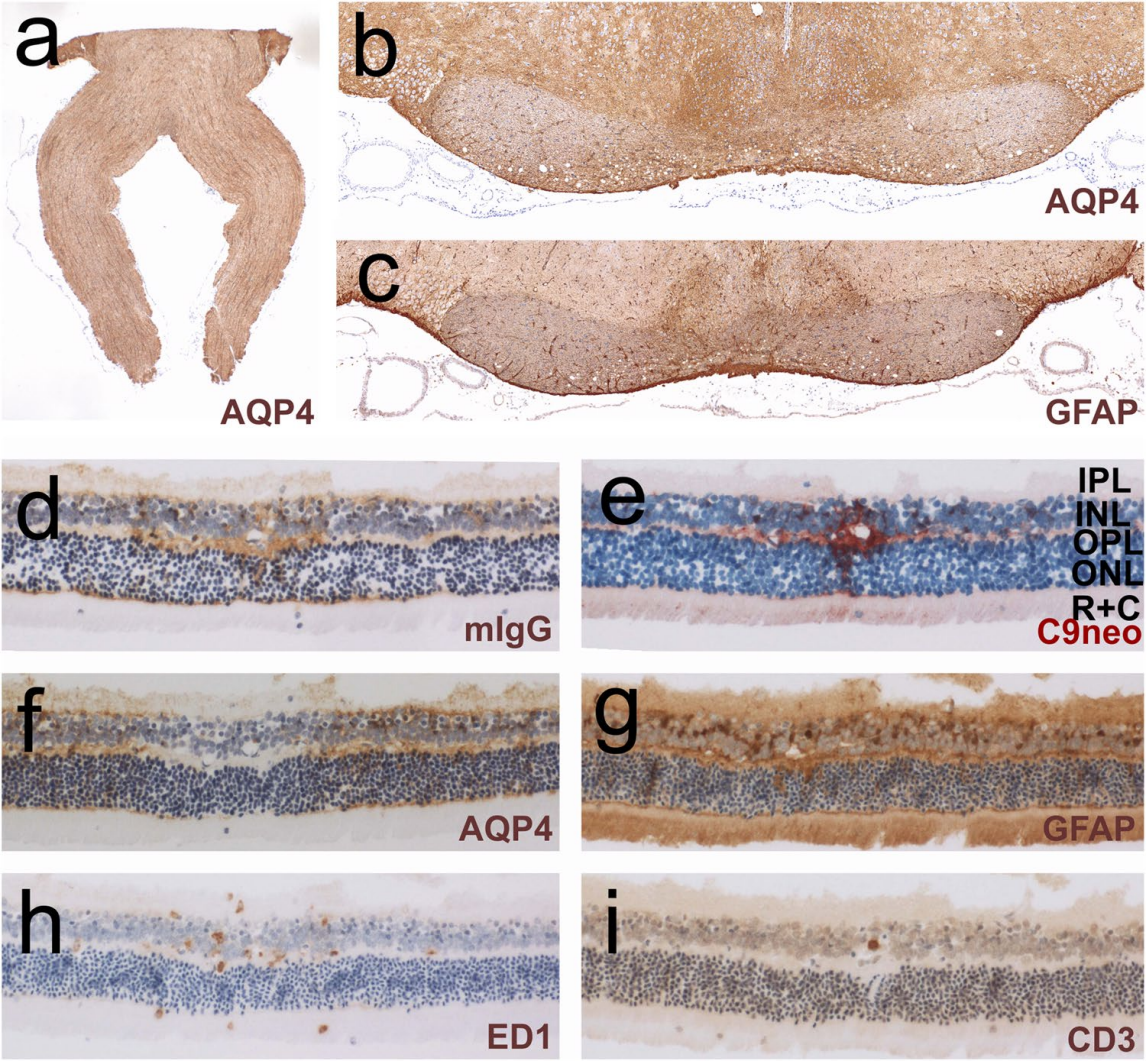
Histological characterization of chiasm, optic nerves, and retina. Lewis rats were injected daily with the monoclonal AQP4-specific murine E5415A antibody and were sacrificed after 120 h (**a**–**i**). The sections were reacted with antibodies against AQP4 (**a**, **b**, **f**), GFAP (**c**, **g**), murine IgG (**d**), complement C9neo (**e**), with the antibody ED1 (**h**), or with antibodies against CD3 (**i**). Positive reaction products are shown in brown (**a**–**d**, **f**–**i**) and red (**e**). All sections were counterstained with hematoxylin to reveal nuclei in blue. *IPL* inner plexiform layer, *INL* inner nuclear layer, *OPL* outer plexiform layer, *ONL* outer nuclear layer, *R + C* layer of rods and cones

We also found ongoing perivascular lesions deep within the CNS parenchyma, in clear distance to other sites of inflammation, and not associated with meningeal lesions (Fig. 4). These vasculocentric lesions with abluminal antibody reactivity formed either independently of the presence of macrophages, neutrophils, or activated microglia, or were associated with single ED1^+^ macrophages or with a small nodule of adjacent ED1^+^ ramified microglia/macrophages as only signs of barrier dysfunction (Fig. 4). We also found perivascular lesions in the retina (Fig. 5).

### The three different modes of antibody entry cause different types of lesions with AQP4 loss

Antibody entry from the area postrema, from meningeal vessels, from Virchow Robin spaces or from small parenchymal vessels unrelated to meninges caused lesions which radically differed from each other in the extent of tissue destruction.

#### Lesions adjacent to the area postrema

The least aggressive lesions were found when AQP4-abs leaked into the area postrema, and spread from this site into the adjacent tissue: there was no (Lewis rats) or only weak evidence for complement/C9neo deposition (RNU rats) on astrocytes, and there were only few ED1^+^, ramified microglia cells at this site. Hence, the molecular or cellular basis for CDC or ADCC was limited. Astrocytes lost their AQP4 reactivity, but survived and continued to express GFAP protein. APP^+^ axonal spheroids/endbulbs indicative for neuronal dysfunction/damage were rarely observed, and there was no evidence for myelin loss (data not shown). CD3^+^ T cells were exceedingly rare or absent (Fig. 2), eosinophils were present in low numbers, and neutrophils were essentially absent (Fig. 2). In Lewis rats, these spreading lesions were clearly demarcated by a border zone formed by ramified ED1^+^ microglia (Fig. 2), reached the floor of the fourth ventricle, and occasionally also the central canal (Fig. 1). In RNU rats, both ramified ED1^+^ microglia and amoeboid ED1^+^ microglia/macrophages were observed in the border zone (data not shown). Very similar lesions were found in a subset of NMOSD patients, which show the loss of AQP4 reactivity in the area postrema and adjacent CNS parenchyma [28]. In addition, in these cases, astrocytes survive and continue to express GFAP, in line with a lack of complement deposits on these cells preventing CDC (Fig. 2).

#### Subpial lesions

In contrast to the benign lesions spreading from the circumventricular organs did the subpial and periventricular lesions contain neutrophils and activated, ED1^+^ amoeboid macrophages/microglia, and were characterized by astrocyte destruction, as evidenced by their loss of AQP4 and GFAP reactivity (Fig. 3). Complement deposits were found well behind the lesion border. Cumulatively, these observations suggest that, in these lesions, ADCC might play a more important role in tissue damage than CDC. There was a sharp border between the lesions and the intact tissue, often running in parallel to the pial surface which suggests that the antibodies entered the tissue from this site (Fig. 3, suppl Figure 3). The meninges above such lesions contained neutrophils, macrophages, and in Lewis rats, also T cells. However, since RNU rats also show severe meningitis and large, subpial/periventricular lesions despite the absence of thymus-derived T cells (Fig. 3), T cells do not play a role in lesion formation. Instead, they are rather indicators for an open BBB allowing the passage of antibodies and cells from meningeal vessels into the underlying parenchyma and into the CSF.

#### Perivascular lesions with AQP4 loss

Perivascular lesions were the most frequent and most aggressive lesions found in brains and spinal cords (Figs. 2, 4, suppl Table 1), and were characterized by massive astrocyte loss (Fig. 4). These lesions contained neutrophils (Fig. 4), some eosinophils, large numbers of ED1^+^ amoeboid macrophages/activated microglia cells, and massive complement deposits throughout the entire lesion area, indicating that both ADCC and CDC contribute to astrocyte damage. Identical lesions are also found in NMOSD medullas [28], for example in the medullas of some patients, where perivascular loss of AQP4 and GFAP reactivity coincides with strong complement deposition (Fig. 2) [10, 25], and in animals with T-cell-induced ENMOSD [35, 36, 57]. However, in marked contrast to their counterparts found in NMOSD patients and in ENMOSD rats, the perivascular lesions in the AQP4 antibody-injected animals were essentially free of T-cell infiltrates (Fig. 4).

A special form of perivascular lesions with AQP4 loss was seen in the retina, where leakage of immunoglobulins and complement components occurred at multiple sites in the outer plexiform layer in 1/5 AQP4-antibody-positive Lewis rats 120 h after the initiation of antibody injections. These lesions contained ED1^+^ macrophages and occasional CD3^+^ T cells indicating a dysfunctional blood-retinal barrier, and displayed loss of AQP4 reactivity by Müller cells, which continued to express GFAP. Since these cells did not show the accentuated surface reactivity of IgG or complement deposits (Fig. 5), AQP4 loss in Müller cells occurred independently of ADCC or CDC, most likely by antibody internalization and degradation, as seen before in ENMOSD [59] and in retinas exposed to AQP4-abs in vitro [8].

### The formation of an initiating lesion in the brain precipitates lesions in the vicinity

**Fig. 6:**
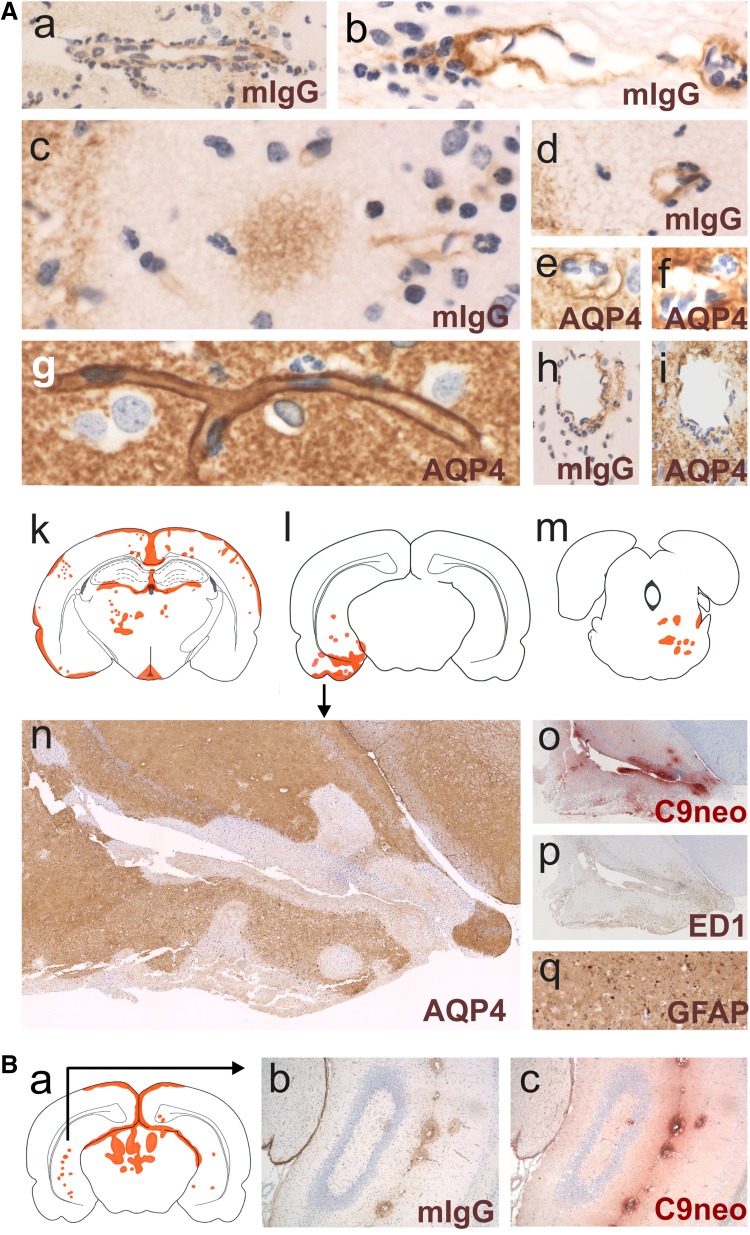
Mechanisms of lesion evolution. **A** Lesions associated with subpial loss of AQP4 reactivity. Shown here are brain sections with blood vessels entering from the meninges, stained with antibodies against murine IgG (**A**a–**A**d, **A**h) or against AQP4 (**A**e–**A**g, **A**i). Positive reaction products are shown in brown. Counterstaining was done with hematoxylin to reveal nuclei (blue). **A**k–**A**m Examples for lesion clusters seen in the brains of individual animals. The lesions were projected in red onto schemes provided by Paxinos and Watson [33] as guide lines. The lesion cluster pointed out by arrow in **A**l was further reacted with antibodies against AQP4 (**A**n), complement C9neo (**A**o), with the antibody ED1 (**A**p), or with antibodies against GFAP (**A**q). Positive reaction products of complement C9neo are shown in red, all others in brown. Counterstaining was done with hematoxylin to reveal nuclei (blue). **B** Example for lesions deriving from branches of the longitudinal hippocampal vein. Shown here are the lesions of a single animal, projected in red onto a scheme provided by Paxinos and Watson [33] as guideline. The lesion cluster pointed out by arrow (**B**a) was further reacted with antibodies against murine IgG (**B**b), and complement C9neo (**B**c)

Most brain lesions were found in clusters (Fig. 6, suppl Figure 1, suppl Table 1). In some of these cases, additional lesions formed in direct continuation with or very close to subpial lesions, for example, via Virchow Robin spaces as described above (suppl Table 1, Fig. 6). In other cases, large vessels and their branches seem to become activated and leaky. For example, in 2/5 RNU rats, several different branches of the longitudinal hippocampal veins [55] became centers of lesions with immunoglobulin and complement deposition followed by AQP4 loss (Fig. 6b, and suppl Figure 1).

Finally, subpial and/or perivascular lesions can grow in size, eventually fuse, and provide the pathological substrate for the formation of large, even necrotic, and astrocyte-destructive lesions in the CNS (Fig. 6, suppl Figure 1).

### In the presence of activated, CNS antigen-specific T cells, monoclonal AQP4-abs cause lesions earlier, and in lower antibody concentrations

**Fig. 7:**
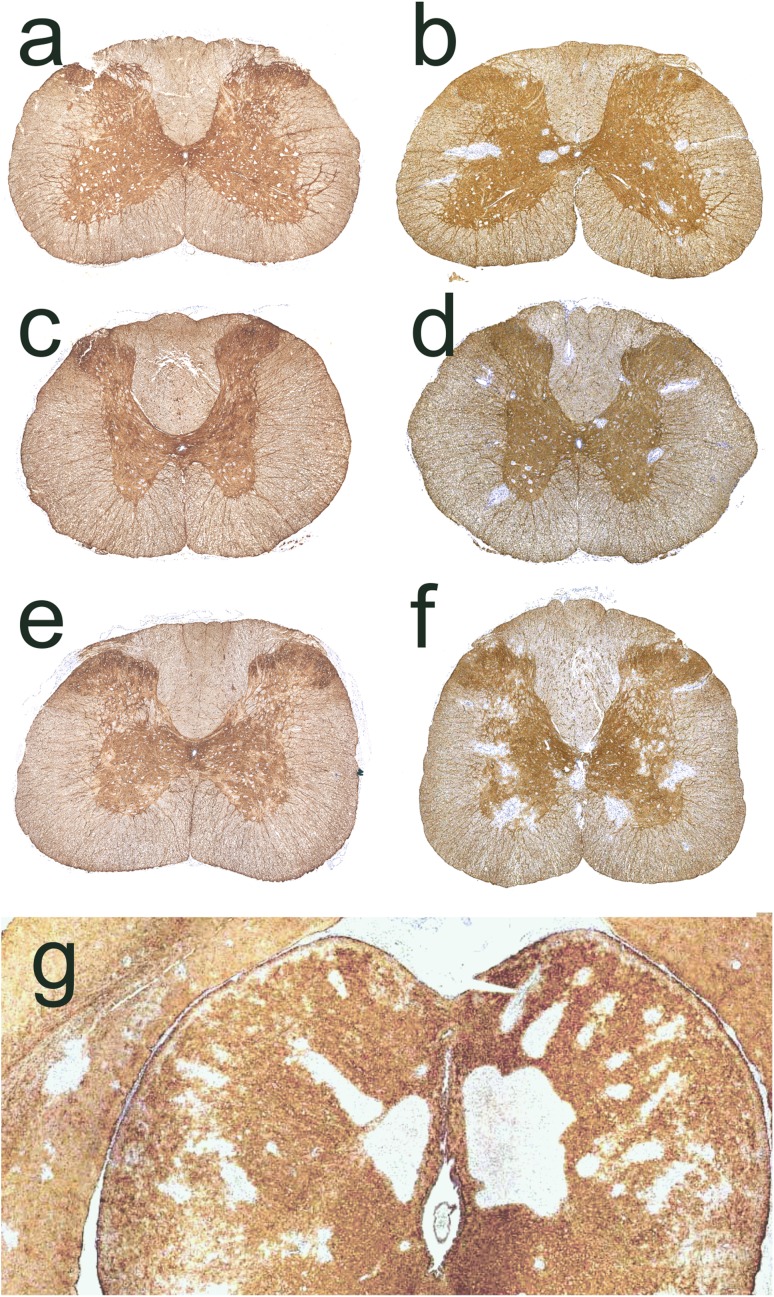
Activated, CNS antigen-specific T cells accelerate lesion formation in the presence of the monoclonal AQP4-specific murine E5415A antibody. Spinal cord (**a**–**f**) and brain (**g**) sections of Lewis rats daily injected with monoclonal AQP4-abs and sacrified 24 h (**a**, **c**, **g**) or 48 h (**e**) after the initiation of antibody injection (**e**), and of Lewis rats which were seropositive for monoclonal AQP4-abs injected once in a concentration of 1 mg (**b**, **d**, **f**) or 0.5 mg (**g**) at the onset of CNS inflammation induced by the activated MBP-specific T cells (**b**, **f**) or AQP4_268–285_-specific T cells (**d**), and sacrificed 24 h (**b**, **d**, **g**) or 48 h (**f**) later. The sections were stained with commercial α-AQP4 antibodies to reveal the expression of AQP4 (brown) and counterstained with hematoxylin to reveal nuclei (blue)

As described above, apart from area postrema lesions, daily injections of 1 mg of AQP4-abs did not induce any perivascular, subpial, or periventricular lesions with AQP4 loss in brains and spinal cords of Lewis rats within 24 or 48 h (Table 1, Fig. 7). However, when the same (1 mg) or even lower amounts of antibodies (0.5 mg) were applied in animals in which CNS antigen-specific T cells opened the BBB to start CNS inflammation, perivascular lesions with AQP4 loss formed within 24–48 h, and even with lower antibody concentrations (Fig. 7, [23]).

### Antibody effects on peripheral tissues

**Fig. 8:**
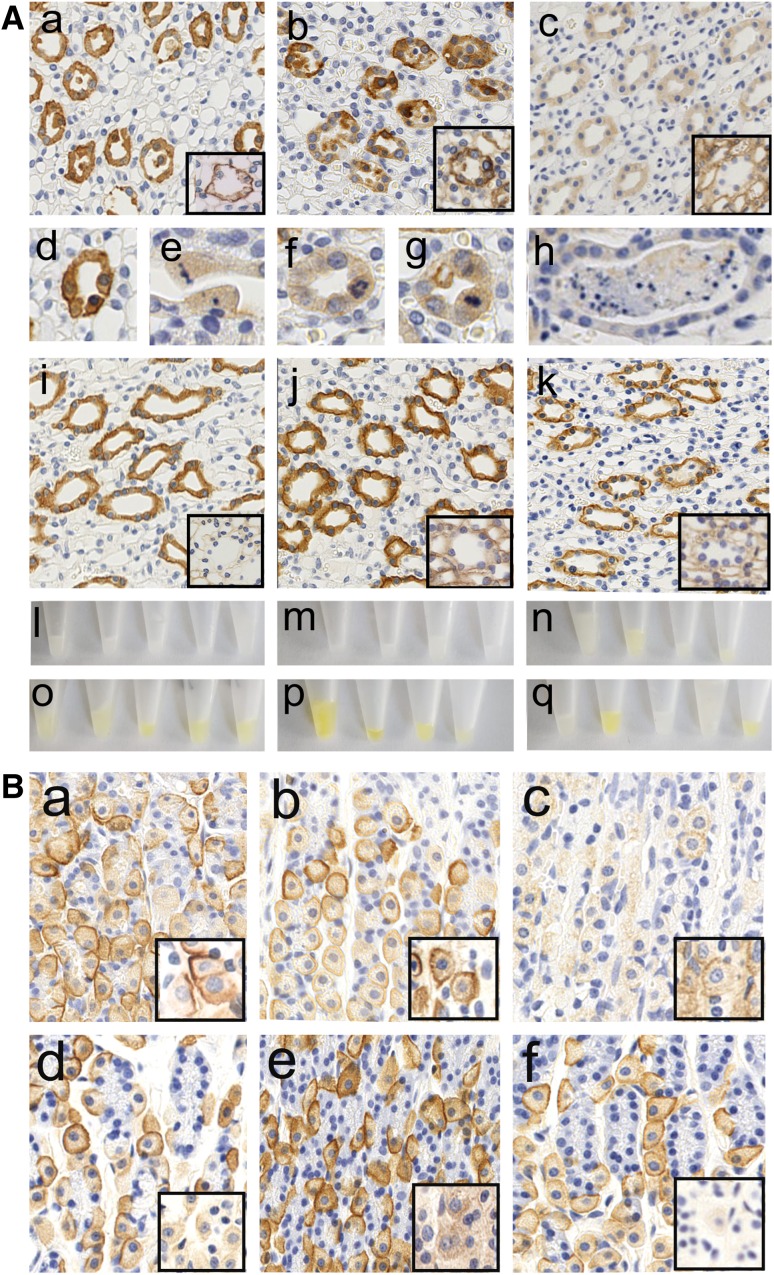
Loss of AQP4 reactivity from peripheral organs. **A** Kidney sections of Lewis rats which had been injected daily with the monoclonal AQP4-specific murine E5415A IgG (**A**a–**A**h) or with murine control IgG (**A**j–**A**k) were analyzed. Tissue was sampled after 0 h (**A**i), 24 h (**A**a, **A**d, **A**e), 48 h (**A**b, **A**f, **A**g, **A**j), and 120 h (**A**c, **A**h, **A**k), and stained with commercial α-AQP4 antibodies to reveal the expression of AQP4 on the cell membrane of kidney collecting duct epithelial cells (brown, large pictures), or stained with antibodies against murine IgG to reveal the binding of the injected murine E5415A antibody to AQP4 on kidney collecting duct epithelial cells, and the lack of binding of the injected murine control IgG (brown, small inserts with black rims) to these sites. Please note that, after 24–48 h presence of E5415A, detached epithelial cells are found within the lumen of the collecting ducts. **A**d–**A**h detailed pictures of kidney collecting duct epithelial cells stained with commercial α-AQP4 specific antibodies. **A**d–**A**e When E5415A was present for 24 h, the epithelial cells show the strong basolateral expression of AQP4, the formation of pycnotic nuclei and ongoing detachment of epithelial cells from the basement membrane. **A**f–**A**g When E5415A was present for 48 h, many epithelial cells show reduced levels of AQP4 expression. The tubular cells are plump and show mitotic figures as evidence for ongoing regeneration. **A**h When E5415A was present for 120 h, rare casts of cellular debris were seen in the lumen of collecting ducts. Urine from Lewis rats injected daily with the monoclonal AQP4-specific murine E5415A IgG was isolated after 24 (**A**l), 48 (**A**m), or 120 h (**A**n) and compared to urine of uninjected rats (**A**o), and to urine of rats daily injected with murine control IgG and collected after 48 (**A**p) or 120 h (**A**q). **B** Stomach sections of Lewis rats which had been injected daily with the monoclonal AQP4-specific murine E5415A IgG (**B**a, **B**c) or with murine control IgG (**B**d–**B**f). Tissue was sampled after 0 h (**B**d), 24 h (**B**a), 48 h (**B**b, **B**e), and 120 h (**B**c, **B**f), and stained with commercial α-AQP4 antibodies to reveal the expression of AQP4 on the cell membrane of parietal cells (brown, large pictures), or stained with antibodies against murine IgG to reveal the binding of the injected murine E5415A antibody to AQP4 on parietal cells, and the lack of binding of the injected murine control IgG (brown, small inserts) to these sites

In the first 24–48 h, the injected monoclonal AQP4-abs bound to the basal side of AQP4 expressing kidney collecting duct cells (Fig. 8). There was no evidence for complement-mediated cell destruction (data not shown). Instead, collecting duct epithelial cells rounded up, detached from the basal membrane, and were shed into the ductal lumen. Some shed cells had pyknotic nuclei (Fig. 8). Rarely, karyorrhexis was seen in the unshed collecting duct epithelial cells (Fig. 8). At the same time, collecting duct regeneration was evidenced by numerous mitotic figures (Fig. 8). Despite these histological changes, the animals produced copious amounts of clear urine (in contrast to the more concentrated yellow urine produced by the relevant control animals, Fig. 8). There was no visible hematuria, no sign of proteinuria (suppl. Figure 4), and no evidence of AQP4-abs in the urine (data not shown). After 120 h, occasional tubular casts were seen, but the cellular detachment and shedding of epithelial cells had essentially stopped. Instead, the collecting duct cells displayed greatly reduced AQP4 expression, and binding of the injected monoclonal AQP4-specific antibody could no longer be detected. Urine color returned to control levels (Fig. 8). AQP4 loss over time was also observed on gastric parietal cells (Fig. 8).

## Discussion

We show here that high affinity AQP4-abs can enter the CNS on their own, and employ different mechanisms for lesion formation and growth.

**Fig. 9:**
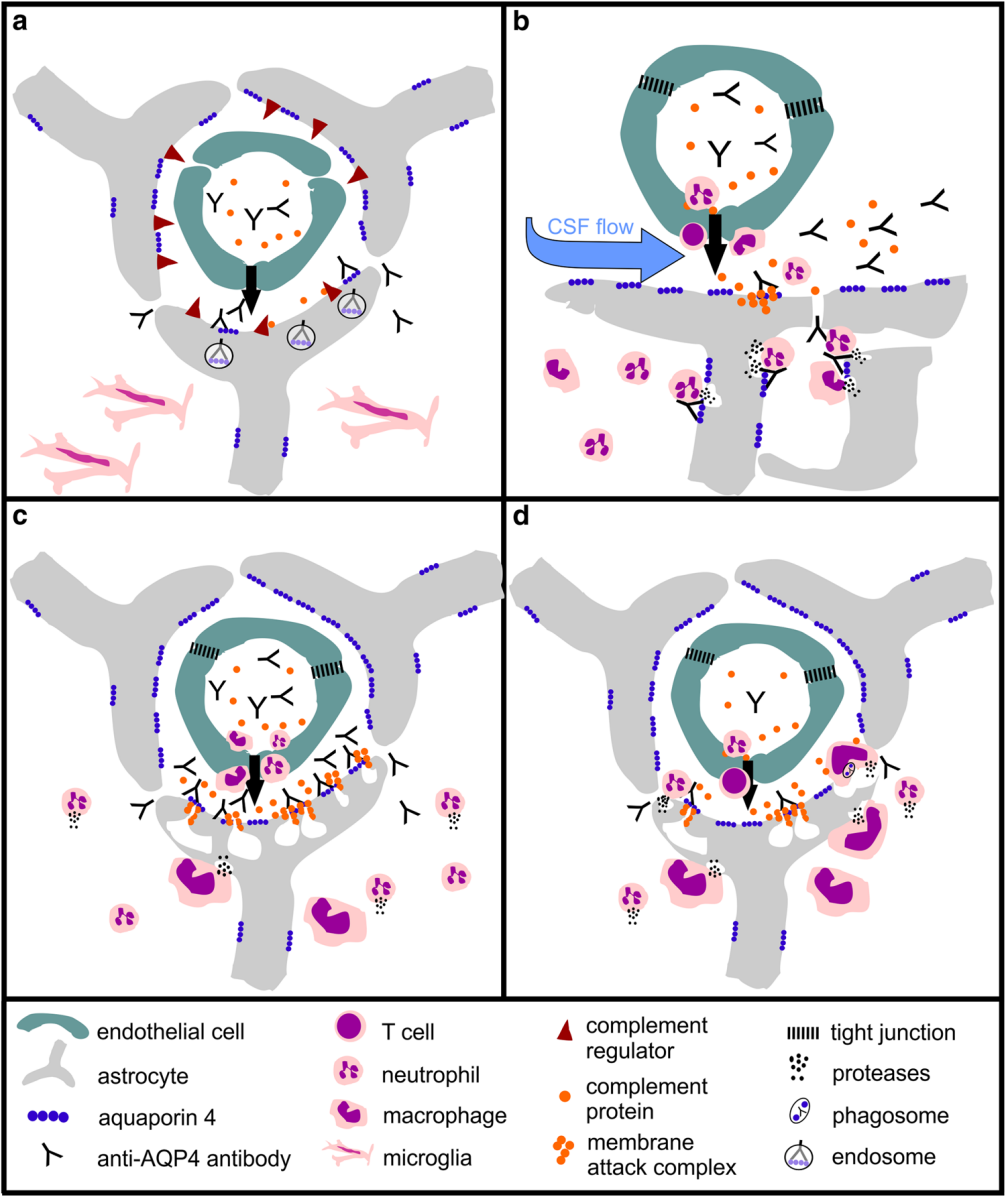
Four possible scenarios of AQP4 antibody-induced tissue damage. **a** Entry through the circumventricular organs. Here, there is just some protein leakage in the absence of major vascular damage. Thus, moderate levels of autoantibodies will induce AQP4 loss, but there is little complement leakage and little recruitment of effector cells, and the astrocytes survive. **b** Leakage through meningeal vessels. Since meningeal vessels are not covered by astrocytic foot processes, AQP4 antibody leakage will not result in amplified damage of the blood–brain barrier. Thus, complement activation will be minor in the subpial areas and needs longer to build up. In addition, complement factors leaking from meningeal vessels will be diluted by CSF and washed away by CSF flow. **c** Leakage through parenchymal vessels. Only by this route, AQP4 antibodies cause massive focal damage to the blood–brain barrier, associated with profound leakage of complement components, which are not washed away due to the narrow extracellular space of the brain parenchyma. **d** When there is a primary T-cell-mediated inflammation, inflammation will be targeted to the meninges and the parenchymal vessels (not present in circumventricular organs). In this case, inflammation induces damage to the blood–brain barrier, thus allowing entry of AQP4-abs, complement proteins, and effector cells, activates effector cells, and also induces local production of complement components, in particular in macrophages. Thus, only rather low concentrations of specific antibodies are necessary to cause damage to the AQP4 expressing astrocytes by ADCC and CDC

The most prominent entry site for AQP4-abs was the area postrema which contains fenestrated capillaries [29], allowing the passage of antibodies and other blood-borne molecules. Here, the antibodies bind to astrocytes and cause loss of AQP4 reactivity, but not astrocyte death. Possibly, local astrocytes are protected from CDC by the expression of complement regulators lost upon contact with endothelial cells of the BBB [41], and from ADCC by the lack of neutrophils and fully activated macrophages (Fig. 9). With time, the AQP4-abs further spread from this site into the area subpostrema and the underlying solitary nucleus. Targeting this site by AQP4-abs may cause symptoms of the area postrema clinical syndrome in NMOSD patients, i.e., intractable hiccups or vomiting/nausea [54], and possibly also the nausea/vomiting-related symptom of excessive salivation [12] observed after 120 h in 1/10 rats. Binding of AQP4-abs to astrocytes in the area postrema has also been described in mice before, but was not followed up over a longer time span [40]. Also in the area subpostrema, the antibodies caused AQP4 loss, but no further tissue damage: There was no or only little complement/C9neo deposition, microglia cells were only weakly activated, eosinophils were very rare, and macrophages, neutrophils, and T cells essentially absent. In line with the low amounts of molecules and cells able to participate in CDC or ADCC, local astrocytes survived, continued to express GFAP, and lost their AQP4 reactivity most likely by internalization and degradation of antibody-bound AQP4.

A more aggressive type of lesion was seen when AQP4-abs derived from meningeal vessels and directly entered the underlying parenchyma, or when the antibodies diffused first from the vessels to the CSF before they reached their targets from the pial surface. Then, the resulting lesions were often delineated by ED1^+^ macrophages, and were characterized by influx of neutrophils and macrophages, and by delayed deposition of complement, possibly due to partial diffusion of complement proteins into the CSF (Fig. 9). Hence, in such lesions, mechanisms of ADCC might be more important for astrocyte destruction than CDC. It remains unclear whether the tissue damage seen in the subpial lesions is only initiated by the binding of AQP4-abs to their astrocytic targets, followed by ADCC and CDC, or whether it is further facilitated by additional toxic factors, for example by proteases released from neutrophils into the CSF. Importantly, subpial lesions formed both in the presence (Lewis rats) and absence (RNU rats) of T cells, suggesting that T cells did not induce lesion formation, but most likely just indicate an open BBB. This would also explain the presence of other cells of the immune system in the CSF, like the plasma cells or neutrophils found in the CSF of some NMOSD patients [2, 17].

The most aggressive lesion type was seen when AQP4-abs entered the CNS via parenchymal BBB vessels. These entry points might locally cause the highest densities of pathogenic antibodies, complement proteins, and macrophages/neutrophils, and could, thus, provide the best micro-milieus for astrocyte destruction by ADCC and CDC (Fig. 9). In this case, one would expect a dominance of perivascular lesions even under suboptimal conditions of antibody concentrations and affinities, as seen to date in all T-cell-induced experimental NMO models using human NMO-IgG [4, 23, 35, 36, 43, 53, 57] (Fig. 9).

At first glance, it seems surprising that antibodies from the circulation may enter the CNS via meningeal and parenchymal vessels, especially since the CNS is shielded from the circulation by the BBB, which restricts the entry of serum proteins, including autoantibodies, into CSF or brain tissue. However, the BBB is not an absolute barrier, but allows the establishment of equilibrium of immunoglobulins between serum and CSF in the range of 1:500–1:1000 [56]. In addition, tracer studies using horse radish peroxidase regularly showed the presence of single vascular segments in the brain with increased permeability for proteins [15, 38]. This minor protein leakage in general is insufficient for the accumulation of pathogenic autoantibodies in the CNS to concentrations needed for tissue destruction. However, antibodies against AQP4, even when they reach the CNS in very small amounts, can disturb the astrocyte/endothelial cell interaction and induce further BBB damage in a self-amplifying process, possibly involving the activation of astrocytes [14, 48].

We also observed antibody leakage from vessels encompassing the blood-retinal barrier. In contrast to the vast tissue-destructive lesions resulting from antibody entry via a dysfunctional BBB, and in contrast to the fully established lesions with complete AQP4 loss from Müller cells seen in human NMOSD retinas [11], the retinal lesions of our AQP4-antibody-injected rats were still small, and showed just ongoing AQP4 loss from Müller cells as previously described in animals with ENMOSD [58]. Possibly, antibody leakage into the retina has a different dynamic than antibody leakage from vessels into brain or spinal cord, causing delayed tissue damage.

In the brain, we frequently observed perivascular lesions forming in close vicinity to fully established lesions; for example, lesions underneath subpial areas with AQP4 loss, clustered perivascular lesions, or lesions originating from branches of single, larger vessels suggesting lesion evolution along vascular structures. The formation of such lesions is probably facilitated by factors secreted from pre-existing lesions. The most likely candidate for such molecules might be vascular endothelial growth factor (VEGF), a molecule released from perivascular astrocytes [1, 18, 27], which drives vascular permeability changes and has been correlated to the formation of longitudinally extensive lesions of NMO patients [46]. At the 120 h point in time, our AQP4 antibody-injected Lewis rats do not show enhanced serum levels for VEGF (data not shown), but this would not exclude the release of this factor into local vessels spanning through heavily inflamed tissue. Alternatively, molecules like interleukin-1 secreted from activated macrophages/microglia could activate endothelial cells of the BBB to produce neutrophil and monocyte recruiting chemokines [21]. Enlargement and subsequent fusion of such clustered lesions could then provide the pathological substrate for the formation of extensive, tissue-destructive lesions [23].

In all, we observed more severe lesions, more lesions in general, and also more subpial lesions in AQP4-antibody-injected RNU rats compared to Lewis rats and NMOSD patients [6, 9, 44]. The most likely explanations for these differences might be the elevated neutrophil counts and the increased macrophage cytotoxicity characteristic for RNU rats [45]. We also observed that RNU rats had lesions more often in hypo/thalamus, striatum, and cerebellum than Lewis rats did, and speculate that this might be due to the different genetic backgrounds of these two strains of rat.

In the current manuscript, we demonstrate that AQP4-specific autoantibodies initiate large lesions in the CNS. Why were such changes not detected in earlier studies? First of all, they used NMO-IgG, an immunoglobulin preparation of NMOSD patients, which typically contains a polyclonal population of AQP4-abs, with different affinities [7]. This is in marked contrast to our study, which used a murine monoclonal AQP4-specific antibody with very high affinity. Then, while human antibodies can interact with rat complement [4], mouse antibodies can do so much better [24], paving the ground for the formation of very large lesions in our antibody-injected animals, which are strikingly similar to lesions also seen in NMOSD patients. Second, many earlier studies employed antibodies in the context of T cell-mediated CNS inflammation [2, 4, 31, 35, 36, 43, 53, 57], and were, for technical reasons, terminated 24–48 h after antibody transfer. Third, AQP4-abs injected into mice once on day 0 were shown to target peripheral AQP4 expressing organs and the medulla, but were removed from the circulation after ~ 48 h, by binding to peripheral, AQP4 expressing organs [40]. Finally, there was just one report about the daily injection of human NMO-IgG into Lewis rats for 4 consecutive days, starting 3 days after their immunization with complete Freund’s adjuvant containing Mycobacterium tuberculosis. The authors showed perivascular loss of AQP4 and GFAP reactivity in the spinal cord of one out of three experimental animals, but then concluded that the lesions were triggered by danger signals provided by non-specific, adjuvant-induced inflammation [20].

As described above, an earlier study demonstrated that AQP4-abs were almost completely cleared from the circulation of mice within ~ 48 h after injection, by binding to AQP4 expressing cells in peripheral organs [40]. Similarly, we observed that, after the first two injections, AQP4-abs bound to AQP4 expressing kidney collecting duct epithelial cells and stomach parietal cells, and we also concluded that the lack of increase in serum titers between the first two injections could be ascribed to antibody clearance mechanisms. However, when the AQP4-abs were present for 120 h in the circulation, a profound increase in titer could be observed, in parallel with a decrease in AQP4 expression in peripheral organs. We ruled out that this decrease was caused by mechanisms involving ADCC or CDC, but did not follow up further whether the decrease in the stomach parietal cells was due to internalization and degradation of antibody-loaded structures, or due to a downregulation in AQP4 expression. In the kidney, however, we could demonstrate that the decrease was associated with a re-organization of collecting ducts, initiated by the shedding of strongly AQP4 expressing cells and culminating in the repair of ducts by epithelial cells with lower AQP4 expression. Apparently, these changes in AQP4 expression in peripheral organs were tolerated by the organism, most likely due to expression of complement inhibitors in peripheral organs preventing CDC [41], due to the low numbers of macrophages found at these sites preventing ADCC, and due to the presence of other AQP molecules compensating for a loss of AQP4 [52]. Hence, over time, AQP4-specific antibodies may change peripheral organs from an antibody-depleting environment to an environment allowing titer increase. It remains to be seen whether an increase in serum antibody titer seen in NMOSD patients is paralleled by changes in AQP4 expression in peripheral organs.

Although we clearly show that AQP4-abs can induce lesions in, otherwise, healthy organisms, provided that they have a sufficiently high affinity and are present over a longer period of time, there are important differences between our experimental model and the human disease which merit attention:

First, we had a somewhat artificial situation in challenging the rats with one single, high affinity and highly pathogenic population of monoclonal AQP4-abs culminating in very high antibody titers. Human NMOSD patients have a polyclonal population of serum antibodies against AQP4 [2, 22, 32, 49], with a mixture of affinities [2], and often with much lower antibody titers [47], which might reduce the efficiency to which their pathogenic antibodies can enter the CNS parenchyma on their own. We speculate that, under such conditions, AQP4-abs seropositive NMOSD patients might even be spared from developing clinically and radiologically overt NMOSD until their AQP4-abs titers become sufficiently high, until high affinity AQP4-abs emerge in their antibody repertoire, or until CNS antigen-specific T cells become activated and open the BBB for the entry of the AQP4-abs into the CNS, as shown in many animals models before [4, 23, 35, 36, 43, 53, 57].

Second, in our experimental Lewis rats, the antibodies were introduced into a normal immune system, while the antibodies in NMOSD patients are found in an immune repertoire with expanded CNS antigen/AQP4-specific T cells [50, 51]. Following their activation, such T cells may open the BBB for antibody entry, allow lesion formation at earlier points in time, under conditions of lower antibody concentrations and affinities, and at more NMO-typical sites like brain stem, optic nerves, and spinal cords [4, 23, 35, 36, 43, 53, 57].

One could also argue that we used a recombinant rodent antibody, and that the ultimate proof whether a patient-derived antibody can access the CNS remains to be demonstrated. We have tested the AQP4-ab E5415A in cell-based assays, as they are used in the diagnosis of NMOSD patients, and found that it recognizes human AQP4, just like patient-derived antibodies do. Moreover, this antibody shows a loop A-dependent binding pattern, as also typical for many human AQP4-specific antibodies (data not shown). The ultimate proof whether a patient-derived antibody is able or not to access the CNS on its own can only be convincingly done in animal models which do not only carry patient-derived antibodies (by transfer or transgenic expression), but also contain human complement proteins and human immune cells, to avoid other critical issues like possible suboptimal interactions between human antibodies and rodent complement affecting CDC, or between human antibodies and rodent Fc gamma-receptor III molecules on neutrophils and microglia/macrophages affecting ADCC. Such a humanized animal model does not (yet) exist.

Our study suggests a number of biomarkers possibly useful for the management of NMOSD patients.

1. It might be helpful to test patients not only for AQP4-abs titers, but also for AQP4-abs affinities. While antibody titers are not predictive of the disease course in NMOSD [19], high antibody affinities might be and could be an indication for the early plasmapheresis.
2. The presence of neutrophils in the CSF could be an important marker for subclinical dysfunction of the blood–CSF barrier. Interestingly, CSF analysis from 89 patients with antibody-positive NMOSD revealed the presence of neutrophils in 49% of cases in relapse, but also in 19% of patients in remission [17]. Perhaps, such patients could profit from a more immunosuppressive treatment.
3. The presence of plasmablasts in the CSF could indicate a continuous local source of antibody production. In fact, the first pathogenic AQP4-abs were cloned from cells found in the CSF of an NMOSD patient [2], and plasma cells were described in the CSF of 30% of NMOSD cases in remission [17]. It might be possible that the presence of plasma cells/blasts in the CSF of NMOSD patients warrants tighter monitoring and more aggressive treatment at the very onset of relapse, to prevent the formation of extensive subpial/periventricular lesions.
4. Testing urine of NMOSD patients for elevated numbers of collecting duct epithelial cells and/or casts might be a non-invasive method to detect ongoing changes in the levels of AQP4 expression in peripheral organs, possibly preceding an increase in AQP4-specific serum antibody titers.

Further studies are needed to evaluate the usefulness of these biomarkers for the management of NMOSD patients.

## Electronic supplementary material

Below is the link to the electronic supplementary material.

Supplementary material 1 (DOCX 14 kb)

Supplementary material 2 (TIFF 16440 kb)

Supplementary material 3 (TIFF 8386 kb)

Supplementary material 4 (TIFF 18095 kb)

Supplementary material 5 (TIFF 4949 kb)

Supplementary material 6 (DOCX 13 kb)
