# Supplementary material for: Circulating AQP4-specific auto-antibodies alone can induce neuromyelitis optica spectrum disorder in the rat
Source: Acta Neuropathol. 2018 Dec 18;137(3):467–85. doi: 10.1007/s00401-018-1950-8 (PMC6514074; doi:10.1007/s00401-018-1950-8)
Supplement: Supplementary file 1 — Supplementary material 1 (DOCX 14 kb) [file 401_2018_1950_MOESM1_ESM.docx]

**Suppl Fig 1: Distribution of lesions with AQP4 loss in the CNS of individual Lewis and RNU rats**

Shown here are brain and spinal cord [cervical (C1–7), thoracal (T1–10) and lumbar/sacral (L1–S4)] sections as well outlines of optic nerve, chiasm, and optic tract of individual Lewis (a, n=5) and RNU rats (b, n=5). The animals were analyzed 120 hours after daily intraperitoneal injections of AQP4-abs, and the location of established lesion with AQP4 loss was projected in red color into the schemes provided by Paxinos and Watson (33) as guide lines.

**Suppl Fig 2: AQP4 loss in the area postrema over time**

Shown here is the mean absence of AQP4 reactivity in % of the total area of the area postrema (+/- standard error of the mean) of Lewis rats injected 1x (24 hrs; n=5) or 2x (48 hrs, n=4) with the AQP4-specific antibody E5415A. As controls, we pooled animals which were injected not at all (n=4) or were injected with murine IgG and sacrificed after 120 hours (n=5). Pooling of these rats was possible, since they did not show statistically significant differences in areas with absent AQP4 reactivity (Mann-Whitney test, p-value (exact, two-tailed = 0.2857). Please note that there is a statistically significant loss of AQP4 reactivity 24 and 48 hours after the injection of E5415A (** p = 0.0014, * p = 0.0469, Kruskal-Wallis test followed by Dunn´s multiple comparisons test).

**Suppl Fig 3: Lesions with AQP4 loss and lesion border running in parallel to pial surface.**

(**a**) Consecutive sections of subpial brain lesions of Lewis rats seropositive for AQP4-specific antibodies (**a**a-**a**c) or mouse IgG (**a**d-**a**f). The sections were reacted with anti-murine IgG (brown, **a**a, **a**d), anti-AQP4 (**a**b, **a**e), and anti-GFAP (**a**c, **a**f).

(**b**) Consecutive sections of subpial spinal cord lesions of RNU rats seropositive for AQP4-specific antibodies (**b**a-**b**c) or mouse IgG (**b**d-**b**f). The sections were reacted with anti-murine IgG (**b**a, **b**d), anti-AQP4 (**b**b, **b**e), and anti-GFAP (**b**c, **b**f).

In all cases, positive reaction products are brown. Counterstaining was made with hematoxylin to show nuclei in blue.

**Suppl Fig 4: No evidence for proteinuria in antibody-injected rats.**

Analysis of urine from animals injected daily with the monoclonal AQP4-specific murine E5415A IgG (anti-AQP4 ab) or with murine control IgG (mIgG). Spontaneously released urine was sampled after 24 (n=5 for both groups), 48 (n=3 and 4 for anti-AQP4 ab and mIgG ab injected animals, respectively), or 120 hours (n=4 and 5 for anti-AQP4 ab and mIgG ab injected animals, respectively), and was analyzed with Combur^5^ Test® HC Test strips (Roche) to determine the presence of protein. Urine of 5 uninjected rats (no IgG) was tested as well. Additional controls were drops of phosphate buffered saline (neg) or of phosphate buffered saline containing 1% rat serum (pos).
