# Supplementary figures and images for: Circulating AQP4-specific auto-antibodies alone can induce neuromyelitis optica spectrum disorder in the rat
Source: Acta Neuropathol. 2018 Dec 18;137(3):467–85. doi: 10.1007/s00401-018-1950-8 (PMC6514074; doi:10.1007/s00401-018-1950-8)

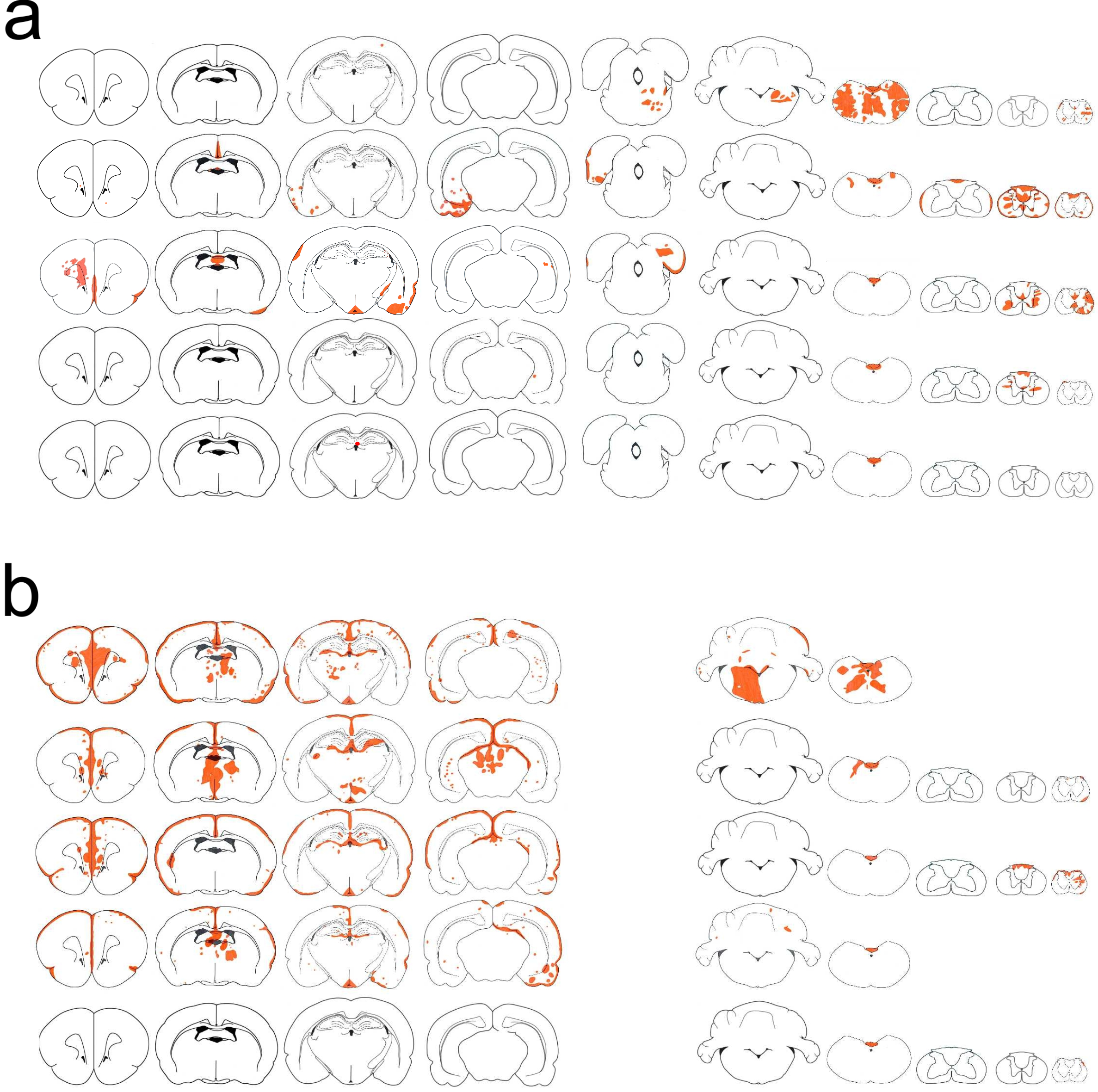

Supplement: Supplementary file 2 — Supplementary material 2 (TIFF 16440 kb) [file 401_2018_1950_MOESM2_ESM.tif]

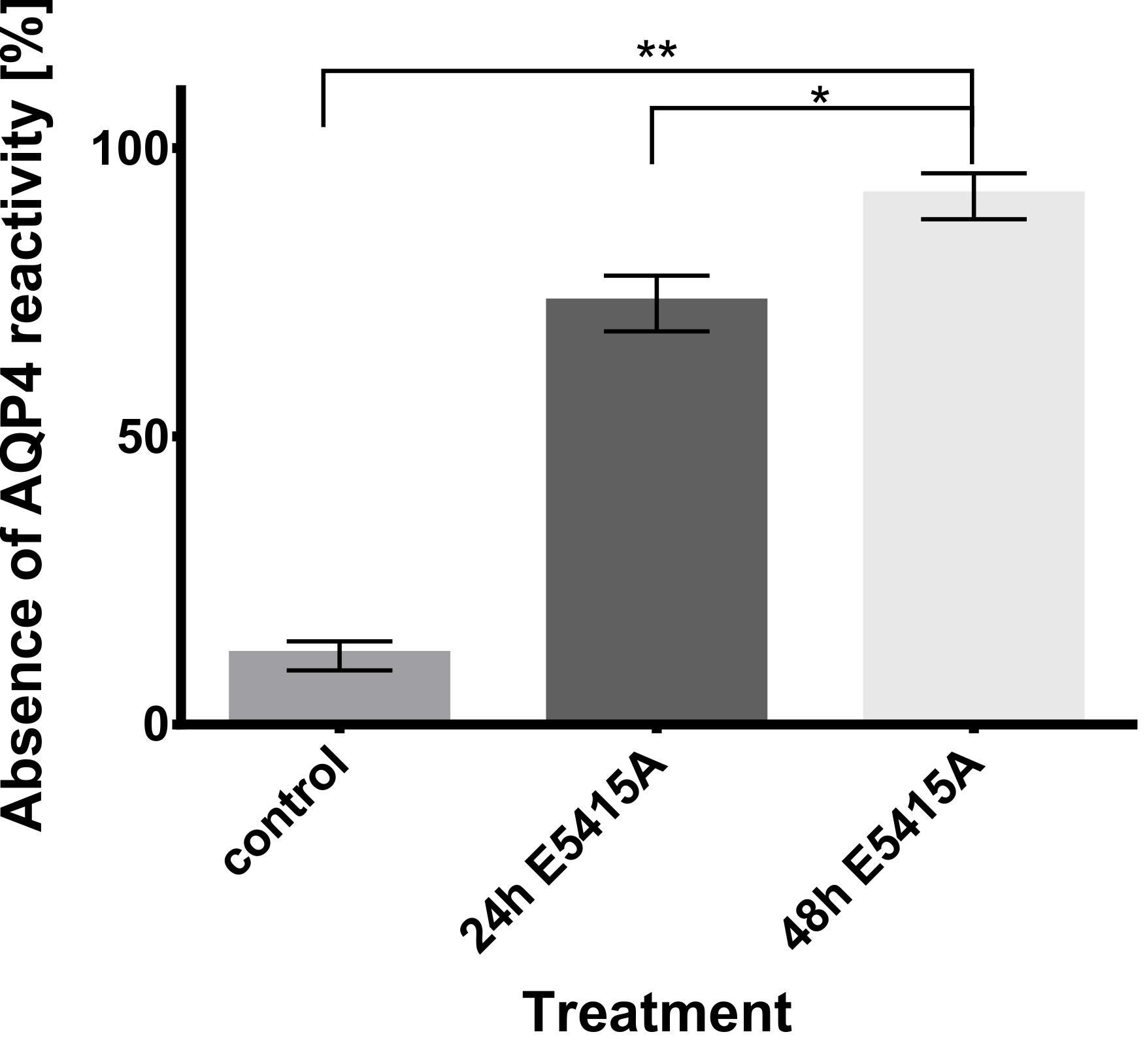

Supplement: Supplementary file 3 — Supplementary material 3 (TIFF 8386 kb) [file 401_2018_1950_MOESM3_ESM.tif]

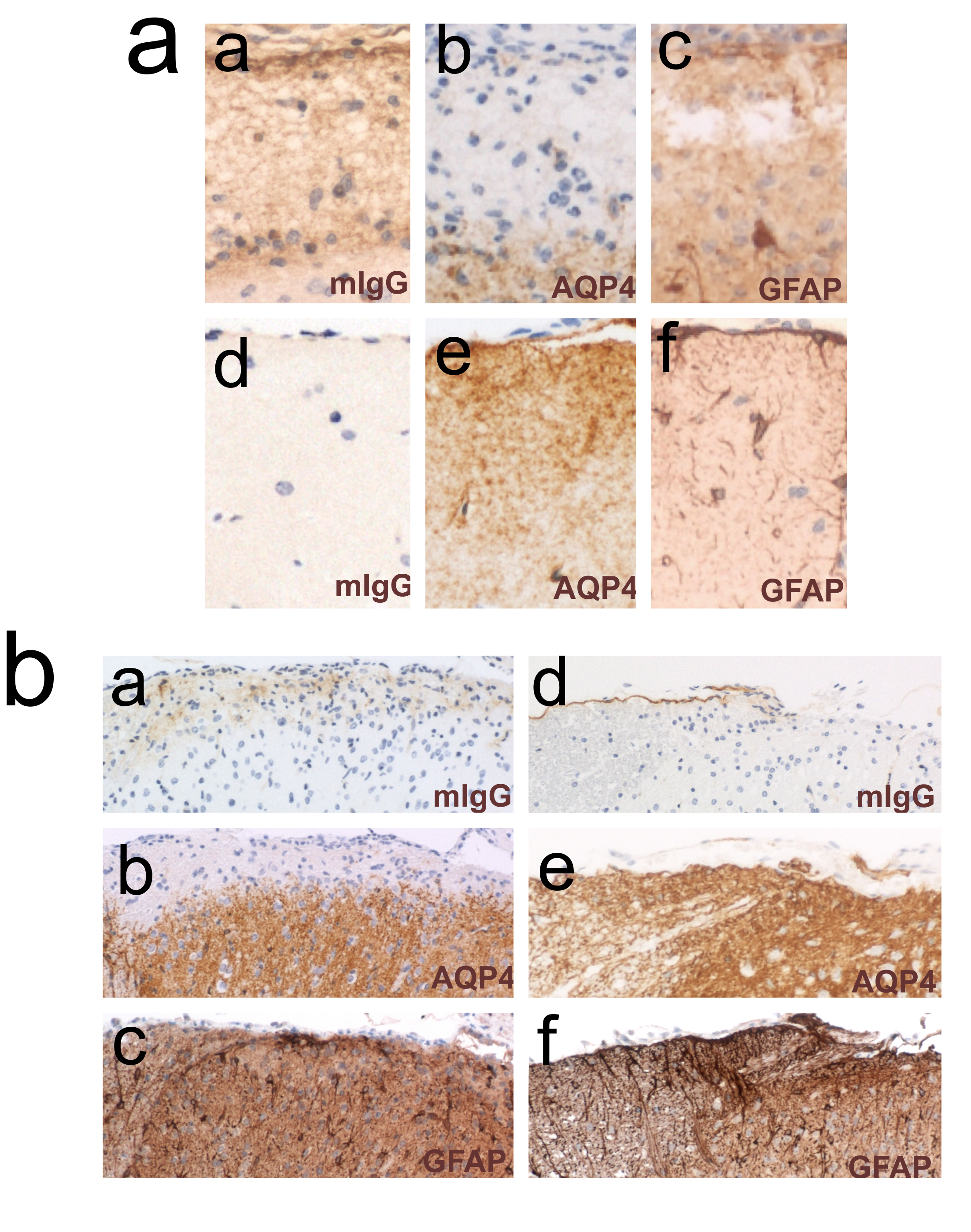

Supplement: Supplementary file 4 — Supplementary material 4 (TIFF 18095 kb) [file 401_2018_1950_MOESM4_ESM.tif]

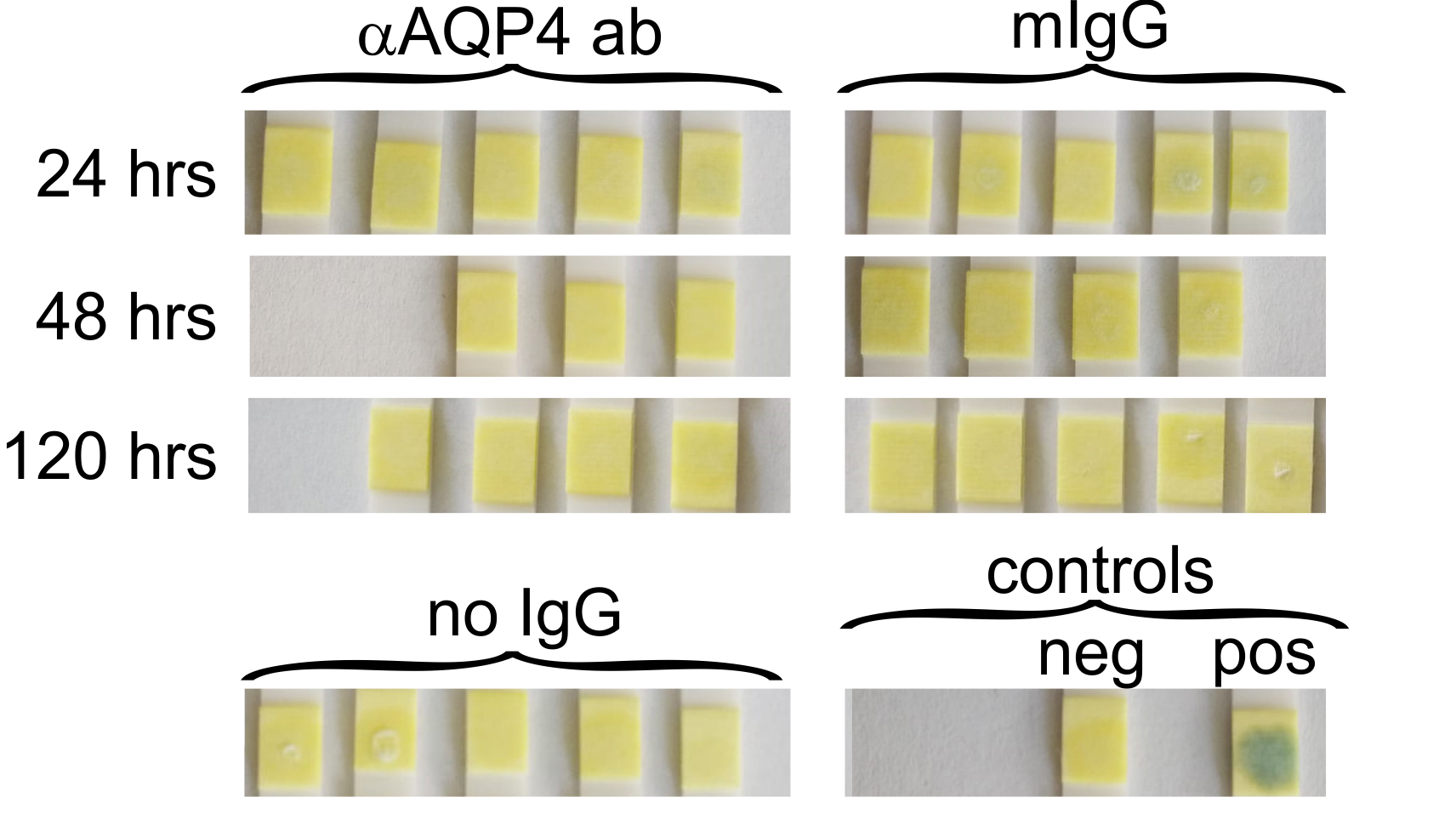

Supplement: Supplementary file 5 — Supplementary material 5 (TIFF 4949 kb) [file 401_2018_1950_MOESM5_ESM.tif]
