# Supplementary material for: Circulating AQP4-specific auto-antibodies alone can induce neuromyelitis optica spectrum disorder in the rat
Source: Acta Neuropathol. 2018 Dec 18;137(3):467–85. doi: 10.1007/s00401-018-1950-8 (PMC6514074; doi:10.1007/s00401-018-1950-8)
Supplement: Supplementary file 6 — Supplementary material 6 (DOCX 13 kb) [file 401_2018_1950_MOESM6_ESM.docx]

**Suppl table 1: Frequencies of different types of established lesions in Lewis and RNU rats.**

|  | Lewis rats | RNU rats |
| --- | --- | --- |
| % of brain lesions in clusters | 81 | 85 |
| % of subpial lesions in the brain | 25 | 16 |
| % of subpial lesions in the spinal cord | 37 | 50 |
| % of perivascular lesions associated with or close to subpial brain lesions | 21 | 24 |
| % of perivascular lesions associated with or close to subpial spinal cord lesions | 26 | 42 |
| % of perivascular lesions in clear distance to subpial brain lesions | 47 | 57 |
| % of perivascular lesions in clear distance to subpial spinal cord lesions | 37 | 8 |
| % of brain lesions with unclear origin | 7 | 3 |
| % of spinal cord lesions with unclear origin | 0 | 0 |
